# How do inner and outer settings affect implementation of a community-based innovation for older adults with a serious illness: a qualitative study

**DOI:** 10.1186/s12913-020-06031-6

**Published:** 2021-01-07

**Authors:** Grace Warner, Emily Kervin, Barb Pesut, Robin Urquhart, Wendy Duggleby, Taylor Hill

**Affiliations:** 1 Associate Professor School of Occupational Therapy, Dalhousie University, P.O. Box 15000, Halifax, NS B3H 4R2 Canada; 2 Mount Saint Vincent University, 166 Bedford Highway, Halifax, NS B3M 2J6 Canada; 3 University of British Columbia Okanagan, 1147 Research Road. Arts 3rd Floor, Kelowna, BC V1V 1V7 Canada; 4 Department of Surgery, Dalhousie University, Rm 8-032, 8th floor, Centennial Building, 1678 South Park St, Halifax, NS B3H 2Y9 Canada; 5 University of Alberta, 3-141 ECHA 11405 87th Ave., Edmonton, AB Canada; 6 Department of Psychology and Neuroscience, Dalhousie University, 6299 South St, Halifax, NS B3H 4J1 Canada

**Keywords:** Community, Older adults, Health system, Organizational factors, Implementation science, Consolidated framework for implementation research, Volunteer navigators, Primary care, Palliative approach to care

## Abstract

**Background:**

Implementing community-based innovations for older adults with serious illness, who are appropriate for a palliative approach to care, requires developing partnerships between health and community. Nav-CARE is an evidence-based innovation wherein trained volunteer navigators advocate, facilitate community connections, coordinate access to resources, and promote active engagement of older adults within their communities. Acknowledging the importance of partnerships between organizations, the aim of our study was to use the Consolidated Framework for Implementation Research (CFIR) to explore organizational (Inner Setting) and community or health system level (Outer Setting) barriers and facilitators to Nav-CARE implementation.

**Methods:**

Guided by CFIR, qualitative individual and group interviews were conducted to examine the implementation of Nav-CARE in a Canadian community. Participants were individuals who delivered or managed Nav-CARE research, and stakeholders who provided services in the community. The Framework Method was used to analyse the data. Particular attention was paid to the host organization’s external network and community context.

**Results:**

Implementation was affected by several inter-related CFIR domains, making it difficult to meaningfully separate key findings by only inner and outer settings. Thus, findings were organized into themes informed by CFIR, that cut across other domains and incorporated inductive findings: intraorganizational perceptions of Nav-CARE; public and healthcare professionals’ perceptions of palliative care; interorganizational partnerships and relationships; community and national-level factors that should have facilitated Nav-CARE implementation; and suggested changes to Nav-CARE. Themes demonstrated barriers to implementing Nav-CARE, such as poor organizational readiness for implementation, and public and health provider perceptions palliative care was synonymous with fast-approaching death.

**Conclusions:**

Implementation science frameworks and theories commonly focus on assessing implementation of innovations within facilities and changing behaviours of individuals within that organizational structure. Implementation frameworks need to be adapted to better assess Outer Setting factors that affect implementation of community-based programs. Although applying the CFIR helped uncover critical elements in the Inner and Outer Settings that affected implementation of Nav-CARE. Our study suggests that the CFIR could expand the Outer Setting to acknowledge and assess organizational structures and beliefs of individuals within organizations external to the host organization who impact successful implementation of community-based innovations.

**Supplementary Information:**

The online version contains supplementary material available at 10.1186/s12913-020-06031-6.

## Background

Evidence has shown that identifying and supporting individuals early in their trajectory toward end of life, such as those who have serious life-limiting conditions, can improve their quality of life, symptom management, mental health, and in some cases, life expectancy [1–3] This is commonly defined as a palliative approach to care [4].

Implementing a palliative approach to care early in the individual’s trajectory toward end of life can necessitate creating partnerships between primary healthcare and community organizations to better support patients and their families [5]. Community organizations can provide non-medical supports to address psychosocial problems that complement medical care [6–8]. The value of linking health services to community supports at end of life has been formally recognized for over 20 years in the health promoting palliative care initiative [9–11]. The health promoting palliative care initiative leverages community resources to make palliative care more self-sustaining [9]. It also promotes creating partnerships between health professionals and the communities within which they practice [12]. Qualitative research has demonstrated that stronger partnerships can increase health providers’ understanding of what services can be delivered by community organizations in conjunction with the healthcare system to improve patient-centred care and continuity of care for patients struggling with serious illness [13, 14]. Although there have been challenges to conducting costly research evaluations such as controlled trials on community-based initiatives [6, 7], the strength of this movement is evidenced by the integration of health promoting palliative care innovations in palliative care services in Australia [15, 16], Scotland [17], and WHO recommendations [8].

Often one of the supports community organizations offer is volunteers that can support patients and families nearing end of life [18]; this extends available resources to enhance quality end-of-life care [19, 20]. In particular, volunteers who support patients and their families dealing with a serious illness can improve access to care and provide psychosocial support in a non-medical context that may be more appropriate than through the healthcare system when these needs are best met through personal, informal relationships [18, 21, 22].

Despite the benefits community-based volunteers offer, there are recognized barriers to volunteers working in conjunction with health providers. Specifically there can be problems with role clarity, role boundaries, and differing views of palliative care between health providers and volunteers [19, 23]. Volunteers’ perspectives on palliative care often align with the palliative approach to care and encompasses the needs of individuals with serious life-limiting conditions, whereas health providers may not consider these individuals to be palliative as their death is not imminent [24].

This study applied the Consolidated Framework for Implementation Research (CFIR; 14), to examine a community-based volunteer program called Nav-CARE (Navigation—Connecting, Accessing, Resourcing, Engaging). Nav-CARE is an evidence-based program that has been evaluated in several communities using mixed methods analysis, it has been found to have a positive impact on the quality of life of older persons living with serious illness [25–32]. The program trains volunteers to provide navigation services to older adults and their families dealing with serious chronic illness [25, 28]. Community-based volunteer navigators complement the role of healthcare professionals by focusing on the daily challenges seniors experience as they progress through the illness trajectory, rather than healthcare navigation [18]. The Nav-CARE volunteer role aligns with the current emphasis on strengthening the role of communities to deliver a palliative approach to care.

There are numerous implementation science theories, models and frameworks [33]. Damschroder et al.’s [34] CFIR was chosen because it reflects an amalgamation of domains and constructs identified in prior studies, and is more appropriate than most frameworks or theories for assessing community and health system-level factors that affect implementation [35, 36]. The CFIR domains and constructs are listed in Table 1.

**Table 1:** CFIR domains aligned with Nav-CARE, and CFIR constructs

**1. Intervention Characteristics, NavCARE Program** o Intervention source o Evidence strength and quality o Relative advantage o Adaptability o Trialability o Complexity o Design quality and packaging o Cost	
**2. Outer Setting, Community and health system where Nav-CARE was implemented** o Patient needs and resources o Cosmopolitanism o Peer pressure o External policy and incentives	
**3. Inner Setting, Hospice organization and administrators trialing Nav-CARE** o Structural characteristics o Networks and communication o Culture o Implementation climate o Readiness for implementation	
**4. Characteristics of Individuals, Nav-CARE volunteers, volunteer coordinator, social worker at hospice** o Knowledge and beliefs about the intervention o Self-efficacy o Individual stage of change o Individual identification with organization o Other personal attributes	
**5. Process, Aspects of delivering Nav-CARE** o Planning o Engaging o Executing o Reflecting and Evaluation	

Evidence from the field of implementation science indicates innovations need to be tailored to the context where they are being implemented [37]. Our study supplemented data from a larger study [38, 39] with stakeholder interviews in a single community to explore context-specific factors affecting successful implementation within that community; it focused on the inner and outer setting domains in the CFIR framework. The rationale for focusing on the inner and outer setting domains was an interest in understanding how organizational support for Nav-CARE and links between organizations affected implementation. In addition, the pre-identification of specific domains and constructs within the CFIR is recommended by Damschroder and colleagues [34]. Although two CFIR domains were pre-identified, CFIR domains often interact [40]. Thus, although the primary aim of this study was to use the CFIR to clarify critical barriers and facilitators at the organizational (Inner Setting) and community or health system levels (Outer Setting) to Nav-CARE implementation the study assessed interactions with other CFIR domains. The research question was: How do the CFIR Inner and Outer Settings interact with other domains to facilitate or hinder implementation of Nav-CARE?

## Methods

A large mixed-methods study that trialed Nav-CARE in eight sites across three Canadian provinces found it benefited older adults [38, 39, 41]. The qualitative portion of this study conducted semi-structured individual and group interviews with participants who had delivered or managed Nav-CARE. The sub-study for this manuscript supplemented data from the larger study with individual stakeholder interviews and additional group interviews with participants who had managed Nav-CARE in one of the community sites to highlight contextual factors that could be transferable to the other locations. The inner and outer setting CFIR domains were pre-identified as being of interest; however, the study considered all CFIR domains during data gathering and analysis.

### Context

Nav-CARE volunteers worked within a community hospice organization in a semi-rural area approximately an hour from the nearest large city. The community population was 12,000, 25% were over the age of 55 and 22% lived unattached. The hospice was a non-profit organization whose mandate was to “support families facing life threatening illness, death and grieving the loss of their loved one” [42].

### Nav-CARE intervention

The Nav-CARE intervention involved a two-day training for Nav-CARE volunteers and the volunteer coordinator; the hospice social worker participated in 1 day of that training alongside the volunteers. The training was provided in-person by an advanced practice nurse who performed a nurse navigator role and a mentor role in the early pilots of the Nav-CARE intervention [32]. Participants were provided with an implementation handbook with key points from the training. After the training, the volunteers and coordinator were offered monthly coaching calls with the advanced practice nurse who provided the original training. Volunteer coordinators were also offered the opportunity to participate in teleconferences every 6 weeks to share implementation experiences.

Once trained, the coordinator role was to oversee day-to-day management of volunteers and help recruit older adults for the project. Day-to-day management involved screening clients and volunteers, overseeing volunteers, and providing support as needed. The social worker’s role was to support volunteers in complex situations where the volunteer felt it was beyond their training and a health professional’s expertise was needed. The role of the volunteer was to visit older adults and their family members (if available) dealing with serious illness to provide support and help them navigate community services. The precise frequency and timing of visits was negotiated with the older adult. In addition, volunteers administered evaluation measurements for the research component of the study. To facilitate client recruitment to Nav-CARE, the volunteer coordinator invited individuals from the community to form an advisory committee when the program was initiated. Four community-dwelling older adults with serious illness were recruited in this setting and Nav-CARE volunteers visited them for 1 year. The hospice organization was paid a small monetary incentive every quarter to compensate volunteers and the volunteer coordinator for their time.

### Participants

Hospice staff included an executive director, a volunteer coordinator, an administrator, and a social worker. Study participants included all hospice staff and Nav-CARE volunteers that were part of the larger study. Staffing changes at the hospice organization during the intervention limited our ability to obtain individual interviews with some staff at the end of the Nav-CARE program who were present during implementation; nevertheless, these changes allowed us to obtain the perspectives of both new and long-time hospice staff. To account for these changes two group interviews were conducted with hospice staff at the end of the project to facilitate sharing of experiences across the project timeline. One group interview was done with the advisory committee, which was comprised of representatives from community-based organizations and health services. For this sub-study additional group interviews were conducted with hospice staff, and individual interviews were conducted with key stakeholders who provided services to older adults and/or those with serious illness in the community. The stakeholders were purposively sampled to provide different perspectives on community capacity for Nav-CARE. As the need for different perspectives was identified during the qualitative process individuals were identified through snowball sampling who could provide that knowledge.

### Interview guides

Individual and group one to two-hour interviews with participants were conducted in-person at a location convenient to the interviewee, or by telephone if distance was prohibitive. A group interview [43] was chosen instead of a focus group, because this format was more conducive to our research. In a group interview there is more direct interaction between the researcher and participants than in a focus group. When a researcher conducts a group interview, they primarily ask questions and probe individual participants. In contract, when a researcher conducts a focus group they have less direct interaction with each participant and participants have more interactions with each other.

The initial interview guides were based on sample interviews available on www.cfir.org and trialed in a prior study [44], then tailored to gather specific information about the Nav-CARE program. Copies of the interview guides are provided in a supplemental file. Table 2 has a list of when each participant was interviewed. The semi-structured interview guide used with two of the hospice staff at implementation midpoint queried experiences with Nav-CARE, supports needed for their Nav-CARE roles, and what was needed to sustain Nav-CARE. Questions for the advisory committee interview, two hospice staff group interviews, and individual stakeholder interviews queried barriers and facilitators to implementation and what was needed to sustain Nav-CARE in the future.

**Table 2:** Participant interviews

Type of participant	Type of interview	Timepoint for Interview
Advisory committee	Group	Nav-CARE beginning of implementation
Hospice Staff	Individual	Nav-CARE mid-point implementation
Hospice Staff	Group	Nav-CARE end of implementation
Nav-CARE volunteers	Individual	Nav-CARE end of implementation
Stakeholders	Individual	Nav-CARE two to eight months post- implementation

One of the key areas probed concerned the slow recruitment of older adults into the Nav-CARE program; it took 9 months to recruit the first older adult. Interview guides were tailored to gather specific information about Nav-CARE using CFIR domains and constructs as probes to identify relevant areas of inquiry.

### Analysis

All interviews were conducted by the first author and the research assistant trained in qualitative interviewing and analysis. Interviews were recorded using a digital audio recording device, transferred from the device following each interview, and transcribed verbatim by an experienced transcriptionist. Following transcription review, interviews were uploaded into NVivo 12. All interviews were de-identified; a code was given to each interview and personal identifiers were stripped from the data.

For this study, CFIR domains aligned with the following facets of Nav-CARE: Intervention characteristics (Nav-CARE program); Outer setting (Community and health system where Nav-CARE was implemented); Inner setting (Hospice organization and administrators trialing Nav-CARE); Characteristics of individuals implementing Nav-CARE (Nav-CARE volunteers, volunteer coordinator, social worker at hospice) and, Process (aspects of delivering Nav-CARE). Although our research question focused on the Inner and Outer Settings, we also assessed how other CFIR domains interacted with the Inner and Outer Settings.

Content analysis, applying a Framework Analysis approach [45, 46] was used during the study to determine the applicability of CFIR constructs. Content analysis categorizes the data into patterns [47]. Framework Analysis [46, 48], also used in a prior manuscript [44], is an iterative process. It involved three analysts reviewing the transcripts and audio tapes multiple times to familiarize themselves with the material and identify initial themes that were credible, reflecting the participants’ intent. As in most qualitative analysis, the analytic process began early, during initial data collection, to help determine when new information was no longer being generated from interviews. Framework analysis is both a deductive and inductive process. The CFIR framework was used to determine deductive codes, additional inductive codes emerged during analysis to develop the final themes. The inductive codes ensured themes reflected the voice and intent of participants. Using NVivo all codes were mapped to transcript sections and decisions on codes were documented to create an audit trail. Relevant transcript text was charted into themes using matrices. The intent of our analysis was to focus on the CFIR inner and outer settings. However, this thematic framework was too narrow. The themes needed to be broadened to reflect other relevant CFIR domains and constructs, and participant specific language. The codes and themes were reviewed by all analysts multiple times to check for potential biases, ensure they reflected participants’ words, and improve the credibility of reviewers’ interpretation. Additional interviews were added when new themes emerged. Enough information power [49] was reached with our sample size. This conclusion is based on the narrow aim of study focusing on the inner and outer setting domains, specificity of experiences and knowledge of participants, application of established implementation science theories, strong quality of the dialogue elicited in interviews by experienced interviewers, and examination of one case rather than a cross case analysis. To guarantee findings conveyed experiences of participants, processes associated with trustworthiness were enacted such as member checking and reflexivity [50]. Member checking involved sharing findings to date with hospice staff during the end of implementation group interviews, and with stakeholders who were probed during their interviews, on how their perspectives may differ or confirm the perspectives of other participants. Reflexivity was practiced throughout the qualitative analysis between the research group to explore the credibility of our themes. Engaging participants with preliminary findings was used to solidify our conclusions and helped foster trustworthiness.

## Results

Study participants included four hospice staff, two Nav-CARE volunteers, six advisory committee members (palliative care physician, lead for church charity, lead for caregiver support organization, health services manager for the health authority and two hospice staff members), and four community stakeholders (two physicians, social worker, coordinator of a community-based program for older adults). We reached out to seven stakeholders in the community and two did not respond to our emails. However, we felt we had adequate interviews to gather enough information power for our study.

Implementation of the intervention was affected by several inter-related CFIR domains, making it difficult to meaningfully separate key findings by one CFIR domain. As such, our findings were organized into five themes reflecting participants’ perceptions of implementing Nav-CARE, informed by the CFIR framework. At the end of each theme is a summary of what CFIR domains and constructs are associated with the theme. The themes were: (1) Intraorganizational perceptions of Nav-CARE; (2) public and healthcare professionals’ perceptions of palliative care; (3) interorganizational partnerships and relationships; (4) community and national-level factors that should have facilitated Nav-CARE implementation; and (5) suggested changes to Nav-CARE. In the quotes, group interviews (GI) are designated as hospice staff-GI or advisory committee-GI. Individual interviews are designated as hospice staff (HS1, HS2), volunteers (Vol1, Vol2), and stakeholders (SH1-SH4) to maintain participant anonymity. At the end of each theme is a summary of how the qualitative findings fit within CFIR domains and constructs identified in Table 1.

### Theme 1: Intraorganizational perceptions of Nav-CARE

Overall, individuals working within the organization (hospice staff and Nav-CARE volunteers) believed Nav-CARE was worthwhile. Nav-CARE volunteers suggested the program could provide seriously ill older adults and their families with support when they were unable to access support from other health providers such as social workers or physicians. One of the Nav-CARE volunteers felt that she could provide information to clients who “couldn’t find much information” (Vol 1). Volunteers also felt they could provide new insight into what the client was experiencing, because they were outside their circle of care: “… sometimes if you have a person who’s looking … from outside the situation, they might come up with a different idea or just see things a little differently” (Vol 2).

Staff suggested incorporating Nav-CARE volunteers into the care of seriously ill older adults could redistribute some of the workload currently borne by healthcare professionals, creating opportunities for them to allocate their time to more complex cases: “… there already aren’t enough social workers. … if we can continue to build this army of really skilled volunteers, that allows the social workers to focus in on more complicated, complex cases and really work as a team.” (HS 2) Furthermore, they felt Nav-CARE volunteer services could enhance access to psychosocial support for the client and their families: “… just having someone else to talk to sometimes other than your caregiver … can be helpful as well …. if you’re thinking [ …] through some difficult stuff, maybe rather than burdening your caregiver with that, then you have the volunteer navigator to kind of talk some of these things through.” (HS 1).

One of the potential benefits of Nav-CARE volunteers was decreasing social isolation by connecting older adults to their communities:

“… when we first meet them [they] are just incredibly depressed and isolated and we're giving them an opportunity to be bridged into the outer community and saying that you're not alone and there's supports available to make you feel better and you don't have to live like this. … And I think Nav-CARE is absolutely fantastic for that and it's affordable.” (HS 2)

Despite the potential benefits articulated by staff, there were also concerns regarding boundary issues between the roles of volunteers and health providers. For instance, hospice staff 2 described that her “… main concern was putting volunteers into those helping positions where there might be a misunderstanding of what their role is.” Similarly, hospice staff 1 raised concerns over the volunteer understanding the limits to their role: “It all comes around to what the boundaries are of the role and where that line is …”.

Comments in Theme 1 are reflective of the Inner Setting, but also reflect the beliefs and self-efficacy of individuals within the organization. Individuals implementing Nav-CARE described the benefits of Nav-CARE, indicating the organization supported intervention implementation (CFIR Inner Setting domain, Readiness for Implementation construct). While there were comments indicating that they felt Nav-CARE would be beneficial to older adults and could reduce the burden on the health system, there was an individual lack of confidence in how well the Nav-CARE intervention trained volunteers (CFIR Characteristics of Individuals domain, Knowledge and belief about the intervention construct, and self-efficacy construct).

### Theme 2: public and healthcare professionals’ perceptions of palliative care

Stereotyped perceptions of palliative care, and hospice in particular, were mentioned by both stakeholders and hospice staff. Participants identified misunderstandings in the community and health sector about who can access hospice and palliative care, when they can access it, and what palliative care entails. For instance, hospice staff discussed a common misconception around when palliative care is initiated: “… there’s definitely a stigma around palliative care. And a misunderstanding about palliative care, that as soon as you’re on it, you’re going to die within a couple of weeks or months ….” (HS 2).

According to participants, the public understands hospice and palliative care to be synonymous with fast-approaching death. As a result, programs associated with hospice could be misconstrued to only be appropriate for individuals likely to die in the near future. In the words of a Nav-CARE volunteer [2], “… it needs to be sort of reframed so that people think of it as a support and not about death.” Stakeholder 1 felt that “reframing” the perception of hospice and palliative care would need to involve “doing a better job educating and educating and educating the community about what we’re talking about [with respect to early palliative care services].” She continued to explain that the bias extends beyond the community involved in this study to public perceptions across Canada; currently there was no acknowledged “fit” for early palliative care in the public’s perceptions of palliative care.

Additionally, healthcare providers seemed to believe palliative care should only be considered in the last months of life. For example, when Stakeholder 4 had new clients labeled as palliative, she assumed that they were only going to be alive a short time “… how long are they going to be with us?” These beliefs were reflected in the attitudes shown toward the Nav-CARE volunteers. When information on Nav-CARE was sent to a clinic for older adults it was assumed to not be relevant: “Honestly, I do remember something coming across my desk about Nav-CARE … when … I saw that it was associated with hospice, I thought, ‘oh, this is related to the palliative care folks so it’s not really for me’” (SH 3).

Individuals working at hospice acknowledged that it was their responsibility to educate the community about the need for early palliative care and the role of hospice. A hospice staff member in the group interview noted the lack of knowledge of palliative care services in the community: “… there’s a lot of people out there that don’t even know that we’re here and what we do ….” This lack of knowledge likely contributed to individuals not accessing Nav-CARE. In addition, study participants felt that even when individuals were aware of what supports were available to help them, such as the Nav-CARE program, they were sometimes hesitant to access them:

“… it can be very frustrating. Because you hear people talking about unmet needs all the time. And when you say, well, here is a service … [it] can help you get steps closer to meeting those needs. And there is a resounding silence.” (Advisory committee GI)

One of the volunteer navigators suggested that, although there was a need for programs such as Nav-CARE, she felt she needed to convince individuals to participate: “I know there’s lots of lonely people out there who are not well and could benefit from a visit, but I don’t know how to go about convincing them that it’s going to be helpful.” (Vol 2).

Hospice staff emphasized that to increase knowledge about the need for early palliative care and the role of hospice they needed to take a leadership role in promoting connections between hospice and other community organizations to reduce fragmented care:

“… it’s really easy for organizations to be siloed, where we don’t communicate with other organizations unless we have similar missions and similar services … it’s kind of really branching out and just trying to connect with partners that we wouldn’t normally connect with.” (Hospice GI)

Theme 2 is associated with CFIR Process, Engaging and CFIR Outer Setting. It reflects the lack of engagement of individuals in the community with Nav-CARE. It also reveals the impact public and health sector attitudes have on the uptake of community-based innovations, uncovering a deficiency in the CFIR framework Outer Setting domain that will be explored more in the discussion section.

### Theme 3: Interorganizational partnerships and relationships

Stakeholder participants confirmed that community readiness for a palliative approach to care would be positively affected if hospice concentrated on educating and building relationships with community partners that delivered services (e.g., pharmacy care) and programs (e.g., recreational centres). In particular, community pharmacists were thought to have valuable relationships with older adults that would help engage them in conversations concerning palliative care: “How do we interface with the older adults?… Pharmacists. I think we really forget the relationship building that pharmacists have with a lot of the older adults” (SH 1). Connecting with community groups that delivered related services, such as a local caregiver support group, was also suggested as a means of improving awareness of Nav-CARE and recruitment: “An established partnership between Nav-CARE and [caregiver support group] could inform older adults in the community about the resources that Nav-CARE offers.” (SH 2).

Building stronger partnerships between hospice and health services was considered important because these partnerships aligned with the idea of a “shared care” model, which is part of a palliative approach to care. As one participant explained, “the more we share it, the stronger it [care] can be” (SH 1). Building partnerships with individuals delivering home care and primary care could increase awareness of the Nav-CARE program and facilitate recruitment: “… I think you’d definitely have to get the [home care] folks on board” (SH 3). In addition, increased awareness in primary care practices about the need to have end of life conversations with their patients might lead to more patients accessing non-medical supports such as Nav-CARE:

“… I think we really need to continue those conversations of creating awareness that health providers have to be having … these difficult conversations and saying, ‘Okay. You have a chronic illness. Eventually, most likely it will be what ends your life, so why don’t we connect you with those appropriate people … who can help hold your hand during this journey … ’” (HS 2).

To facilitate client recruitment to Nav-CARE, an advisory committee had been established when the program was initiated. At that time, committee members saw the utility of partnering across sectors to support implementation of the Nav-CARE program: “… I would say to make something like this … sustainable, would be having solid partners … where you have a number of different partners who have come together to support and facilitate this particular piece” (Advisory committee GI). Despite the group’s initial enthusiasm for supporting the program, their commitment waned over time and no one was recruited through advisory committee connections: “I reached out to our advisory committee … multiple times and you know, we never received a … a single referral …” (HS 1). Hospice had to resort to other strategies to boost recruitment by posting announcements about the program in the local newspaper and over the radio, and directly contacting primary care practices.

Primary care providers were targeted as critical avenues for patient referrals because they are usually the first point of contact for someone with a serious illness: “that’s usually who you go to first when you’re having a problem” (Hospice GI). Hospice staff felt that “family doctors have a lot of power” (Hospice GI) and “influence” (Hospice GI). However, there was little buy in from primary care providers: “… I think was the biggest challenge in getting those referrals of patients …” (Hospice GI).

Possible factors contributing to low buy-in from primary care may have been providers not understanding what role volunteers had in the circle of care for older adults with serious illness or a fear that they might lose control over the care of their patient: “… I think what happens is healthcare professionals get very protective of the patients” (SH1). Providers also may have been hesitant to involve volunteers in their patients’ care because volunteers had no professional qualifications: “In the healthcare system, qualifications are what earn you your place and your position … a volunteer would be viewed in the same way ….” (SH 3).

The stakeholders interviewed understood how Nav-CARE fit within the circle of care for older adults with serious illness: “… a community volunteer would bring a different perspective that I think would be better in that way. It kind of de-medicalizes it … You know, there’s things you don’t tell your doctor or your nurse that you would certainly tell a close friend or a trusted support.” (SH 3). They felt recruitment would take off once health providers in the community understood the Nav-CARE volunteer role: “...if you can get one primary physician coming in and supporting this and seeing the value of it, I think that’s going to be your biggest selling point…” (Social Worker in Health Jurisdiction).

Despite hospice staff acknowledging that it was their responsibility to increase community awareness of a palliative approach to care and its alignment with Nav-CARE, the hospice members responsible for Nav-CARE did not aggressively push for referrals: “… looking at your referral system is an important piece. Who’s going to take the leadership of that?” (SH 1). This was partially due to “the individuals who were involved” who had not “really carried that ball very successfully and didn’t do the legwork.” (SH 2).

Theme 3 reflects the CFIR Outer Setting domain and how critical it is for organizations implementing community-based programs to establish strong inter-organizational relationships to establish trust or at least familiarity, which seems to be a precursor to other organizations referring clients (CFIR Outer Setting Domain: Cosmopolitanism). There also needs to be strong intraorganizational leadership to support the significant investment of time and energy from individuals involved in creating these networks (CFIR Inner Setting Domain: Readiness for Implementation). Finally, it is essential that staff leading innovative programs actively market the program to other organizations. This finding aligns with the CFIR Process domain, Engaging construct that identifies the need for implementation leaders within the organization and external change agents outside of the organization.

### Theme 4: community and national-level factors that should have facilitated Nav-CARE implementation

There were additional factors within the community and nationally that hypothetically should have enabled partnerships between hospice and health services organizations. First was the recent development of resources, guidelines, and training to help implement a palliative approach to care in primary care, led by a national organization called Pallium Canada. Members of this organization had recently come to the community to lead a workshop with the hospice organization on the compassionate community movement. Also, the health jurisdiction was “trying to build capacity with local physicians and medical staff through [training materials on using a palliative approach to care developed by Pallium], to try and encourage that upstream [approach]” (Hospice GI).

Secondarily, the national creation of laws regarding medical assistance in dying (MAiD) prompted conversations in the media about the future of palliative care and stimulated the public to think more broadly of palliative care versus MAiD. However, the initial surge in public awareness did not evolve into in-depth conversations about what needed to be done to support better palliative care: “So MAiD happened, and then we stopped talking about it” (Hospice GI).

Finally, the high proportion of older adults in the health jurisdiction was expected to lead to an increase in the need for services and supports for older adults with life limiting illness: “… the number of deaths in [the health jurisdiction] are going to triple …. chances are our resources probably aren’t going to be there …” (Hospice GI). This anticipated lack of resources, such as not enough primary or palliative care services, could have facilitated uptake of Nav-CARE as an alternative mode of support; however, some participants perceived it had the reverse effect. Specifically, the lack of services made it harder for primary care providers to think about referring patients to Nav-CARE “… it’s making it hard to do things beyond, you know, that day-to-day helping those people kind of immediately in front of you.” (SH 2).

Theme 4 demonstrates that although there may be factors in the CFIR Outer Setting domain, in the community, that seem to support implementation, such as patient needs for better access to services (CFIR Outer Setting domain: Patient Needs and Resources) or policies facilitating awareness of a palliative approach to care (CFIR Outer Setting domain: External Policy and Incentives), these factors do not always guarantee implementation readiness in the organization or CFIR Inner Setting domain.

### Theme 5: suggested changes to Nav-CARE

Several participants suggested the following modifications to improve client experience and access to Nav-CARE. Volunteers felt clients’ comfort level would increase if they spent less time completing paperwork and more time engaging with the client. Secondarily, although hospice staff thought the initial training was valuable they felt volunteers needed more time and practice: “It could’ve been so much longer than just a couple of days” (HS 2). Nav-CARE had offered volunteers and the volunteer coordinator the option of attending monthly coaching calls and teleconferences to share experiences, but they were very poorly attended. Only one volunteer attended the coaching calls, and the volunteer coordinator did not attend any of these opportunities until the last few months of implementation.

There were also suggestions for expanding Nav-CARE. In the research study, adults with dementia were excluded. However, one participant proposed that adults in the early stages of dementia would benefit from Nav-CARE: “… I mean we certainly don’t meet the needs of those people” (SH 3). It was also suggested that Nav-CARE may be valuable to adults of any age dealing with a chronic condition: “… some people have chronic disease … who would benefit from that navigation support” (SH 2). Alternative sites to hospice that might increase client accessibility were the local Seniors Health Clinic, as well as churches: “there’s a very strong group [of older adults] that attends the church that does a lot in the community ….” (Vol 2). Although the clinic sounded like a viable alternative, the Seniors Health Clinic stakeholder felt there was “some value to not exclusively … being associated with … a medical organization.” He explained the potential advantage of “gain [ing] access to a number of people that don’t have access to those resources” (SH 3).

Theme 5 describes adaptations that could facilitate client access to and experiences of Nav-CARE, that are essential to successfully implementing the program. The thematic elements align with the CFIR Process domain: Reflecting and Evaluating. The process domain cuts across the other four CFIR domains.

## Discussion

The aim of this study was to use the CFIR to clarify critical organizational (Inner Setting) and community and health system (Outer Setting) barriers and facilitators to implementing a volunteer navigator intervention. Although the aim of this study was to clarify barriers and facilitators to implementing a volunteer navigator intervention, the findings did not highlight any strong facilitators. The Nav-Care intervention incorporated processes intended to facilitate implementation, such as the establishing an advisory committee, paying a monetary incentive to the hospice organization to compensate volunteers and the volunteer coordinator for their time, and providing opportunities to get ongoing coaching and support. However, these processes did not appear to have a strong impact on uptake of the intervention.

One of the key findings was the strong influence the CFIR Outer Setting construct Cosmopolitanism had on Nav-CARE implementation. This construct assesses the degree to which an organization is networked or has relationships with other external organizations. Creating relationships increases familiarity and trust between organizations and individuals working within the organizations [9]. In this study, the lack of established relationships between primary care and hospice was likely a contributor to primary care providers’ poor understanding of Nav-CARE, and more generally, a palliative approach to care. Not being familiar with a palliative approach to care is a known barrier to identifying and addressing patients’ palliative care needs early in their trajectory toward end of life [51]. Creating new connections and improving communications between organizations would enhance community awareness of, and readiness for, implementing a palliative approach to care within the community and improve referrals to Nav-CARE.

One way to improve the accuracy of the public’s understanding of a palliative approach to care may be to involve the healthcare providers and practitioners with whom they interact on a day-to-day basis. For instance, community pharmacists have a substantial role in managing the health and well-being of older adults in the community but may be under-utilized [52]. Given the valuable relationships that community pharmacists have with older adults in the community [53], as recognized by the participants in this study, recruiting them as information-providers to share resources with their patients could be a feasible way to promote a palliative approach to care.

Additional barriers related to provider attitudes included a lack of understanding of the role volunteers could play in the circle of care, and not feeling confident volunteers know their role boundaries. Providers’ fears that volunteers may over step their role boundaries is an issue previously noted in the literature [54]. An additional contributor to negative attitudes toward volunteers is that primary care providers traditionally have limited contact with volunteers caring for individuals at end of life so they may not understand what support volunteers could provide to older adults with serious illness [55]. Lack of relationships between organizations combined with negative provider attitudes contributed to poor Nav-CARE recruitment. If Nav-CARE volunteers are to be sustainably implemented, there is a continued need for relationship building to advance the role of volunteers in delivering a palliative approach to care in conjunction with healthcare professionals.

The slow referral to Nav-CARE was likely affected by hospice organizational processes and the lack of commitment to implementing Nav-CARE. The stakeholders’ opinion was that hospice staff needed to do a better job actively promoting Nav-CARE and working with the advisory committee. Hospice staff leading the program may not have had the commitment, nor the right leadership skills, to actively market the program to primary care. Although there seemed to be support for Nav-CARE implementation, hospice staff expressed a lack of confidence in Nav-CARE training and volunteer abilities that seemed to outweigh the benefits they attributed to the intervention. This seemed to translate into an overall lack of organizational readiness within hospice to implement Nav-Care. This may have restricted staff time and resources dedicated to the intervention, such as keeping in regular contact with the advisory committee. This was likely one reason for the volunteer coordinator’s poor attendance at coaching sessions or teleconference support sessions. These findings confirm evidence from the implementation science literature, and specifically CFIR, that there needs to be a formally appointed implementation leader within the organization who actively champions the innovative program [34]. Moreover, they corroborate barriers identified in a scoping review on implementing patient navigation programs [56].

External influences that could have improved participant recruitment to Nav-CARE did not seem to have an effect. National initiatives such as education for health providers on a palliative approach to care and media coverage of MAiD did not seem to bolster recruitment. In addition, although community based support can be an acceptable method of addressing non-pathological distress [57] and provide a relative advantage to health systems, recruitment in primary care was low. Also public perceptions that services delivered by hospice are synonymous with death [58] may have made older adults reluctant to access Nav-CARE. Alternatively, older adults may have been hesitant due to personal factors, such as whether or not the program fit into their daily routine or was appealing to them [59].

Stakeholders’ suggested adaptations to the Nav-CARE program need to be considered in future iterations. Adaptations to volunteer training could reinforce role boundaries, improve volunteer confidence, and enhance development of volunteer-client relationships. Co-locating Nav-CARE with healthcare services might facilitate access.

There were limitations to our study. The narrow focus of our research question and setting for the study may decrease the transferability of our findings to other contexts; however, we provided a description of the community to enable an understanding of our context. Also this narrow focus increased the information power of our study. In addition, we did not conduct member checking with all participants, which may have limited the credibility of our findings. The member checking that was conducted did not change our findings. Furthermore, having three team members involved in coding, an experienced researcher doing the interviews, and confirming our insights in subsequent interviews mitigated the impact of reduced member checking.

## Conclusions

Using CFIR provided a useful structure for uncovering Inner and Outer Setting factors affecting implementation of the Nav-CARE program. Although other domains were important, such as individual attitudes, our study concentrated on constructs within the Inner and Outer Setting domains. The most important Inner Setting construct affecting implementation was readiness for implementation. However, the Outer Setting surfaced as critical. In particular, the Outer Setting construct Cosmopolitanism was key. Our findings suggest, and the literature supports [60], that relationships between community-based organizations are essential to supporting integrated community-based palliative care. More generally, these networks are necessary to sustainably implement community-based programs, especially when programs need client referrals from other organizations [61–63].

Unfortunately most implementation science frameworks and theories focus more on assessing implementation of innovations within facilities rather than in communities [36]. Some frameworks do assess Outer Setting factors [33], but often they have no further details than what is captured in the CFIR [64–67]. If the focus of our study had been different (e.g., to identify strategies for facilitating change) we might have chosen an alternative framework [66, 67]. Nevertheless, implementation frameworks still require adaptation to better assess implementation of community-based programs. One suggestion is to expand the CFIR Outer Setting domain to assess organizational structures and beliefs of individuals within organizations external to the host organization. The CFIR domains and constructs remain valid and useful, but recent literature needs to be explored then consolidated into the framework, to ensure community setting suitability. The community-development literature is one area that could help inform improvements to the framework.

## Supplementary Information

**Additional file 1.**

## Data Availability

The datasets generated and/or analyzed during the current study are not publicly available for privacy reasons but are available from the corresponding author on reasonable request.

## Abbreviations

CFIR: Consolidated Framework for Implementation Research
Nav-CARE: Navigation—Connecting, Accessing, Resourcing, Engaging
GI: Group Interview
Vol: Volunteer
HS: Hospice Staff
SH: Stakeholder
