# Supplementary material for: How do inner and outer settings affect implementation of a community-based innovation for older adults with a serious illness: a qualitative study
Source: BMC Health Serv Res. 2021 Jan 7;21:42. doi: 10.1186/s12913-020-06031-6 (PMC7792161; doi:10.1186/s12913-020-06031-6)
Supplement: Supplementary file 1 — Additional file 1. [file 12913_2020_6031_MOESM1_ESM.docx]

**Interview Guides**

**Stakeholder Interview Questions**

First tell me a little about yourself

1. What organization do you work for?
2. What services or supports does your organization provide to older individuals who have serious illness and their families?
3. How do these services fit with the mandate of the organization?
4. What is your role in delivering, or helping others who deliver, services or supports to older individuals who have serious illness and their families?

Now some information on NavCare

*NavCare involves the provision of navigation services, wherein trained volunteer navigators advocate; facilitate community connections; coordinate access to services and resources; and promote active engagement within communities for older adults and their families dealing with serious illness as they near end of life. NavCare volunteers facilitate connections with community resources to improve quality and continuity of care for this vulnerable population. The NavCare training took place with the Volunteer Coordinator and Volunteers who worked with Colchester East Hants Hospice Society. We are now looking at what factors affect the integration of NavCare services into the Truro community.*

*NavCare involves the provision of navigation services, wherein trained volunteer navigators advocate; facilitate community connections; coordinate access to services and resources; and promote active engagement within communities for older adults and their families dealing with serious illness as they near end of life. NavCare volunteers facilitate connections with community resources to improve quality and continuity of care for this vulnerable population. The NavCare training took place with the Volunteer Coordinator and Volunteers who worked with Colchester East Hants Hospice Society. We are now looking at what factors affect the integration of NavCare services into the Truro community.*

Do you have any questions or comments on the content, design and of NavCare?

1. Can you think of an older adult in your community who might benefit from the services provided by NavCare volunteers?

*Probes:*

- 1. *Suggest examples of older adults who might benefit*

1. Would the NavCare volunteer supplement the services provided by your [insert organization or name of community e.g. primary care or continuing care]?

*Probes:*

- 1. *Do you think the current level of support for those with serious illness who might be in the last year of life is adequate?*
  2. *Is there a need to improve current services for these individuals and their families?*
  3. *Do you think your community perceives a need for services to be improved?*

1. What would be needed for your [insert organization or name of community e.g. primary care or continuing care] to connect your clients/patients to NavCare?

*Probes:*

- 1. *Resources… can be money, training, education, physical space, and time*
  2. *Do you trust the services provided by volunteer organizations?*
  3. *Does communication or coordination with and between organizations need to be improved?*
  4. *What barriers or opportunities exist for [insert organization e.g. primary care or continuing care] in terms of referring clients to NavCare volunteers?*
  5. *How could [insert organization e.g. primary care or continuing care] most effectively be involved with NavCare?*

1. How would NavCare fit with other services and supports in your community?
   1. *Does your community have the infrastructure to support a program like NavCare?*
2. What are possible barriers/facilitators to implementing NavCare in your community?

*Probes:*

- 1. *Does communication or coordination with and between organizations need to be improved?*
  2. *Are there limitations due to policy? Bureaucracy?*

1. Who in the community, your organization, the health district or the province needs to support the NavCare program in order to connect it with health and social services?

*Probes:*

- 1. *Do you feel that your community is empowered to improve palliative care?*
  2. *Think of different individuals and organizations such as would primary care practices or volunteer organizations in your community support this program?*
  3. *Would primary care practices in your community support a volunteer navigator?*

1. How could the community be engaged to support a program like NavCare?
   1. *What kind of education would have to occur for community to support NavCare?*
   2. *What kind of leadership or collaboration would be necessary?*

**Advisory Group Interview Questions**

Thank you for your support for this study. As you may know, our objective is to understand your perceptions of the barriers and facilitators to implementing NCARE*.*

- Can you think of an older adult in your community who might benefit from NCARE?

*Probes: Suggest examples of older adults who might benefit*

- Does the NCARE fit with your priorities, resources and plans?

*Probes: Do you think the current level of support for those in the last year of life is adequate?*

- What resources would be needed in the community/organization to implement the NCARE into current services?

*Probes:*

- *Resources can be money, training, education, physical space, and time*
- *Another type of resource is a provincial, regional or community policy or strategy in the community that supports what this program is trying to achieve?*
- *How would N-Care fit with other services and supports in your community?*
- What three factors do you think will most determine the success of NCARE?
- What are possible barriers to implementing NCARE in your community?
- Who in the community, your organization, the health district or the province needs to support the NCARE in order to make it sustainable?

*Probes:*

- *Think of different individuals and organizations such as would primary care practices or volunteer organizations in your community support this program?*
- *Would primary care practices in your community support a nurse navigator?*
- *Would local volunteer organizations in your community train and support NCARE volunteers?*
- Do you have any comments on the content, design and of the NCARE program?

**Hospice Staff Individual and Group Interview Guide**

- Tell us about your experiences with the NCARE program?
- What has been helpful to you in your role?
- Who or what have supported you?
- How did you find your communication with volunteer navigators? With clients? What changes in communication would you recommend if any?
- How do you feel about the degree of preparedness that volunteers had for the role they were asked to play. What changes would you recommend, if any?
- What do you feel are the most significant contributions NCARE has made to older adults in the community? To their caregivers? Give specific examples if you can.
- What kinds of recommendations would you have for other communities who would like to try this model of navigator services?
- What recommendations would you make for enhancing/changing the program?
- As you think about sustaining this program, what would be your recommendations?
- What have we not asked about, that is important?

**Nav-CARE Volunteer Interview Guide**

- Why did you decide to become an NCARE volunteer?
- Can you begin by telling us generally about your experiences as a volunteer navigator? What was doing this role like for you?
- What did you like most about your NCARE volunteer work?
- What did you like least about your NCARE volunteer work?
- Can you think of any particular barriers or challenges that you encountered in your work as a volunteer navigator?
- What do you feel are the most significant contributions your navigator work has made to older adults in the community? To their caregivers? Give specific examples if you can.
- What kinds of recommendations would you have for other communities who would like to try this model of navigator services?
- What things did you find you were doing that you hadn’t thought you would be doing?
- How about the types of things you thought you would be doing, but you did not do?
- In addition to the time spent visiting clients, how much time did you spend outside of direct visits, e.g., finding resources in the community (this question excludes time spent on the research process such as filling out research paperwork)?
- Will you continue to visit your assigned clients? If this program is made available through your hospice will you continue on in the role? Would you recommend the role to other volunteers? Please explain?
- What recommendations would you make for enhancing/changing the program?
- As you think about sustaining this program within hospice societies, what kinds of things do we need to think about?
